# Anterior leg ulcer secondary to great saphenous vein incompetency: A case report

**DOI:** 10.1016/j.ijscr.2025.111263

**Published:** 2025-04-04

**Authors:** Pezhman Kharazm, Alireza Aghili, Arian Khademi, Alireza Siroosi, Nazanin Musapour

**Affiliations:** Clinical Research Development Center, 5 Azar Hospital, Golestan University of Medical Sciences, Gorgan, Iran

**Keywords:** Chronic venous insufficiency, Greater saphenous vein incompetency, Chronic leg ulcer, Venous ulcer, Duplex ultrasound, Venous mapping, Case report

## Abstract

**Introduction and importance:**

Chronic venous insufficiency (CVI) is a common medical problem causing complications like limb edema, pain, and ulceration. Effective management requires accurate diagnosis and targeted treatment, with Doppler ultrasonography playing a crucial role in assessing reflux pathophysiology and guiding treatment decisions.

**Case presentation:**

A 54-year-old man with a chronic ulcer in his left leg was diagnosed with stasis ulcer. Despite multiple treatments, his ulcer remained active for about 2 years. DUS revealed severe reflux along the greater saphenous vein to a point below the knee. He was scheduled for surgery after a 2-month course of compression therapy. The wound healed after 2 months, and the 6 months' follow-up was unremarkable.

**Clinical discussion:**

Venous ulcers are chronic wounds caused by venous insufficiency. The most common location for ulcers is the peri malleolar region. Doppler ultrasonography is the primary diagnostic tool for managing chronic venous insufficiency, and comprehensive mapping of the entire reflux pathway is essential for optimizing patient outcomes and minimizing the risk of recurrence. Compression therapy is the main treatment for chronic venous insufficiency, and removing the reflux pathway is the most appropriate intervention to enhance wound healing and prevent ulcer recurrence. Less invasive interventions, such as thermal and chemical ablation, and stripping, have become more popular in recent decades.

**Conclusion:**

Although most of ulcers secondary to greater and lesser saphenous vein insufficiency occur in distinct locations, but detailed ultrasonographic mapping of the reflux pathway is critical for prompt management of the venous insufficiency and prevent recurrences.

## Highlights

• In United States, 4 % of adults have venous ulcers.
• Most of ulcers secondary to GSV incompetency occur around medial malleolus.
• Accurate description of reflux pathway is crucial for proper management of venous ulcers.
• Doppler ultrasound is the most useful modality for delineation of the reflux pathway.
• Elimination of reflux is the key factor to treat venous ulcers and prevent recurrences.

## 1 Introduction

Chronic venous insufficiency (CVI) is a common medical problem in general population of developed societies [1]. From the pathophysiologic point of view, CVI is the result of venous hypertension and is associated with significant complications, such as limb edema, pain, and ulceration, leading to a considerable disease burden on patients and healthcare systems alike [2]. There are some known risk factors for CVI, including age, sex, obesity, prolonged standing, and familial predisposition. The effective management of CVI necessitates an accurate diagnosis of the underlying reflux pathophysiology and a targeted approach to correct factors contributing to venous hypertension [3]. Among various diagnostic tools, Doppler ultrasonography plays a pivotal role in assessing venous reflux and guiding treatment decisions. During DUS, the etiology of venous hypertension (thrombosis or valvular incompetency), and the anatomy of the venous hypertension (highest point of reflux and reflux pathway) are delineated [4,5]. In this article, we present a case of chronic leg ulcer secondary to chronic venous insufficiency, emphasizing the management strategy employed and the long-term outcomes of the intervention.

This study is reported in line with the SCARE Criteria [6].

## 2 Case presentation

The patient was a 54-year-old man presented to the clinic with the complaint of chronic ulcer in anterior aspect of his left leg from 2 years ago. He had experienced of ulcer enlargement despite several local and systemic medications. Also, as a construction worker, he had severe pain, swelling and heaviness of his limb, especially on prolonged standings during working. Unfortunately, venous disease was not suggested as the potential cause of this ulcer in any of previous prescriptions. The costs of the wound care had disappointed him as a construction worker with a low economic income from receiving an effective intervention. His past medical history was not significant and he did not remember any relevant trauma on his limb. and he denied substance abuse in any form.

**Fig. 1:**
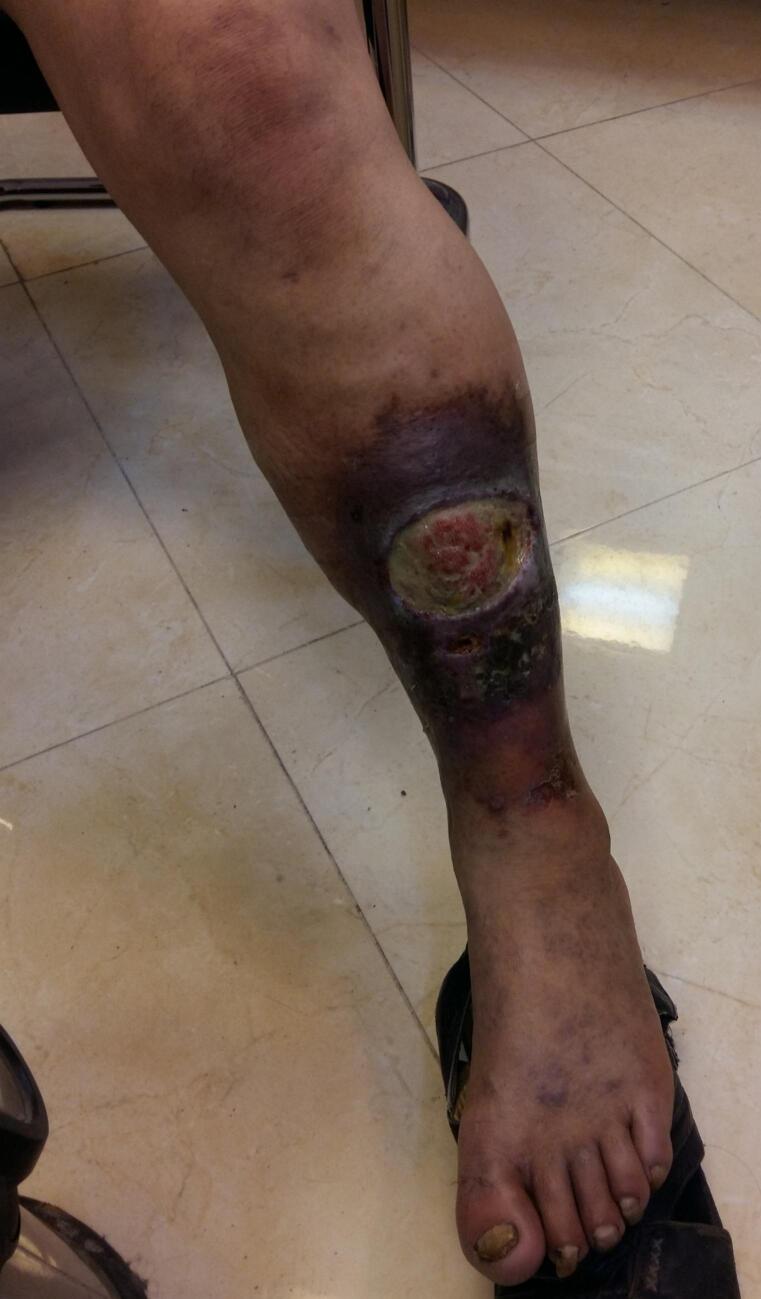
Chronic wound in antero-lateral aspect of left leg. Hyperpigmentation is visible around the wound.

On physical examination vital signs were normal. There was a 7 ∗ 7 cm wound in anterolateral aspect of left leg. The wound had regular margins, well-granulated, red and wet bed with moderate purulent discharge. Hyper pigmentation and lipodermatosclerosis was visible surrounding the wound limited to anterior circumference of the leg. No varicosity was visible on his limb, but reticular veins were visible around his ankle and dorsum of foot. The left leg was mildly warmer than right one but no sign of systemic infection including fever was present. Other points of physical examination were unremarkable (Fig. 1).

With the presumed diagnosis of stasis ulcer, the patient underwent a venous DUS on his affected limb. DUS revealed severe reflux along greater saphenous vein (GSV) from saphenofemoral junction (SFJ) to a point 3 cm below the knee, where the reflux was transmitted to an anterior superficial branch and terminated in a perforating vein connecting to anterior tibialis vein above the wound. The perforating vein had a diameter of 5 mm and seems incompetent on provocative maneuvers. The GSV had moderate tortuosity and diameter of 14 to 17 mm along its course. No signs of acute or chronic thrombosis or reflux were detected along deep veins.

Compression therapy using elastic garment in conjunction with wet to dry dressing for wound care were recommended for the patient. Considering economic limitations of the patient and restrictions secondary to economic sanctions made these options as the only available tools before the surgery was considered. Although the patient reported some decrease in limb heaviness and pain, but the wound remained active after two months.

**Fig. 2:**
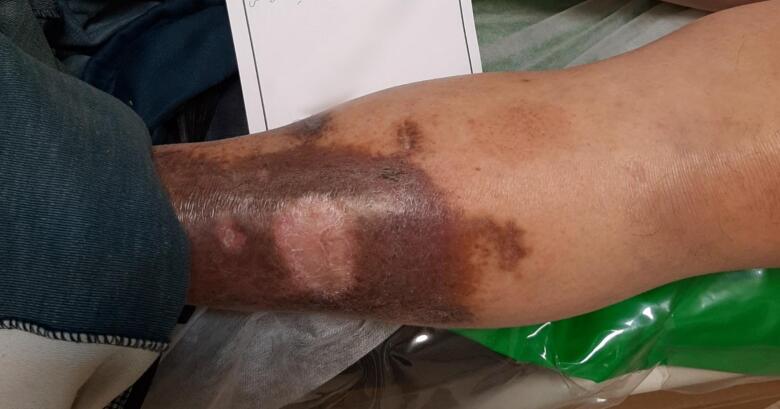
Healed ulcer 6 months after treatment.

The patient was candidate for surgery after a 2-month-course of compression therapy. During surgery, the involved segment of GSV was stripped from SFJ to the aforementioned branch below the knee, and the incompetent perforating vein was ligated at the level of fascia through a separate incision. Post-operative period was unremarkable and compression therapy and wet-to-dry wound dressing was continued for about 2 months. The wound was healed spontaneously after 2 months. The patient was followed for 6 months post-operatively. The skin was intact, the lipodermatosclerosis was resolved and the hyperpigmentation was reduced significantly on the last visit (Fig. 2).

## 3 Discussion

Venous ulcers or in other words, stasis ulcers are chronic wounds that occur secondary to venous insufficiency. About 20 % of adults in the United States have some degree of chronic venous insufficiency, and venous ulcers have a prevalence of 4 % in adults 65 years or older [7]. The ulcer occurs in the location of highest venous pressures according to reflux pathophysiology. The most common location of such ulcers is *peri* malleolar regions, also known as the gaiter area [8]. Our case was a middle-aged man with an ulcer in the anterolateral aspect of his left leg.

Doppler ultrasonography serves as the primary diagnostic tool in the management of chronic venous insufficiency. It is imperative to map the entire reflux pathway comprehensively. Addressing only the initial reflux point may lead to recurrence and continued venous insufficiency through collateral pathways. Thus, thorough evaluation and intervention targeting the complete reflux pathway are essential for optimizing patient outcomes and minimizing the risk of recurrence in chronic venous insufficiency [9,10]. Although most of ulcers secondary to reflux along the greater saphenous vein occur around the medial malleolus, transmission of reflux and resultant venous hypertension to other branches can cause ulceration in other locations [11,12]. In this study, duplex venous mapping revealed the reflux pathway, starting from sapheno-femoral junction, continuation along the great saphenous vein until 3 cm below the knee, transmission to a superficial branch, and at last, draining to anterior tibialis vein through a perforating vein just above the ulcer.

Compression is the mainstay of treatment in all cases of chronic venous insufficiency, and a course of compression therapy is recommended before any intervention [13,14]. In this case, we tried a 2-month course of compression therapy using elastic stockings. Although the patient experienced some improvements regarding pain and heaviness of the limb, but the ulcer remained active.

According to the guidelines, when the pathway of the reflux as the cause of the ulcer is clarified, elimination of the reflux pathway is the most appropriate intervention to enhance wound healing and prevent ulcer recurrence [15]. Saphenofemoral ligation, thermal and chemical ablation, and stripping are available interventions to address reflux along great saphenous vein. Less invasive modalities have become more popular in recent years, although stripping is still recommended for some patients with tortoise and dilated saphenous veins [16]. In our case, we chose stripping and perforating vein ligation, mostly because of economic limitations. The result of intervention was acceptable and no sign or symptom attributable to venous insufficiency was seen in a period of 6 months' follow-up.

## 4 Conclusion

Given the anatomical structure of the lower limb and the drainage of blood from the outer ankle into the small saphenous vein, it is anticipated that venous ulcers on the lateral aspect of the leg result from small saphenous vein insufficiency. However, in this patient, who had no indications of varicose veins, subsequent evaluation with Doppler ultrasound revealed the presence of reflux in the great saphenous vein. Long-term outcomes in the treatment of ulcers necessitate thorough investigation and identification of the underlying venous reflux pathways. Duplex ultrasound is crucial, with a positive sourcing test providing evidence for ulcer venous origin.

## Consent

Consent was obtained for the publication of this article, as well as images from the patient.

## Ethical approval

This study was approved by the Golestan University of Medical Sciences Research Ethics Committee with the following ethics code:

https://ethics.research.ac.ir/IR.GOUMS.REC.1403.411

Date of approval: 2025-02-02

## Guarantor

Dr. Pezhman Kharazm

## Funding

There is no source of funding for this article.

## Author contribution

Pezhman Kharazm: Conceptualization, Writing, Supervision, Editing.

Alireza Aghili, Writing of original draft, Literature review, Editing

Arian Khademi, Writing of original draft, Literature review.

Alireza Siroosi, Writing of original draft, Literature review.

Nazanin Musapour, Writing of original draft, Editing.

## Conflict of interest statement

No conflict of interest is present between authors.

## References

[1] Prochaska J.H., Arnold N., Falcke A., Kopp S., Schulz A., Buch G. (2021). Chronic venous insufficiency, cardiovascular disease, and mortality: a population study. Eur. Heart J..

[2] Sen C.K. (2021). Human wound and its burden: updated 2020 compendium of estimates. Adv. Wound Care.

[3] Naser M., Naser M.M., Shehata L.H. (2022).

[4] Thorisson H.M., Pollak J.S., Scoutt L. (2007). The role of ultrasound in the diagnosis and treatment of chronic venous insufficiency. Ultrasound Q..

[5] Eberhardt R.T., Raffetto J.D. (2014). Chronic venous insufficiency. Circulation.

[6] Sohrabi C., Mathew G., Maria N., Kerwan A., Franchi T., Agha R.A. (2023). The SCARE 2023 guideline: updating consensus Surgical CAse REport (SCARE) guidelines. Int. J. Surg..

[7] Rabe E., Guex J., Puskas A., Scuderi A. (2012). Epidemiology of chronic venous disorders in geographically diverse populations: results from the Vein Consult Program. Int. Angiol..

[8] Sarkar P.K., Ballantyne S. (2000). Management of leg ulcers. Postgrad. Med. J..

[9] Allegra C., Antignani P., Carlizza A. (2007). Recurrent varicose veins following surgical treatment: our experience with five years follow-up. Eur. J. Vasc. Endovasc. Surg..

[10] Yılmaz S., Peköz B.Ç., Dincer N., Deniz S., Oğuzkurt L. (2021). Classification of reflux patterns in patients with great saphenous vein insufficiency and correlation with clinical severity. Diagn. Interv. Radiol..

[11] Labropoulos N., Giannoukas A.D., Nicolaides A.N., Ramaswami G., Leon M., Burke P. (1995). New insights into the pathophysiologic condition of venous ulceration with color-flow duplex imaging: implications for treatment?. J. Vasc. Surg..

[12] Stücker M., Moritz R., Altmeyer P., Reich-Schupke S. (2013). New concept: different types of insufficiency of the saphenofemoral junction identified by duplex as a chance for a more differentiated therapy of the great saphenous vein. Phlebology.

[13] Protz K., Heyer K., Dissemond J., Temme B., Münter K.C., Verheyen-Cronau I. (2016). Compression therapy–current practice of care: level of knowledge in patients with venous leg ulcers. JDDG. J. Dtsch. Dermatol. Ges..

[14] Ratliff C.R., Yates S., McNichol L., Gray M. (2016). Compression for primary prevention, treatment, and prevention of recurrence of venous leg ulcers: an evidence-and consensus-based algorithm for care across the continuum. J. Wound Ostomy Cont. Nurs..

[15] Farah M.H., Nayfeh T., Urtecho M., Hasan B., Amin M., Sen I. (2022). A systematic review supporting the Society for Vascular Surgery, the American venous forum, and the American vein and lymphatic society guidelines on the management of varicose veins. J. Vasc. Surg. Venous Lymphat. Disord..

[16] Juhani A.A., Abdullah A., Alyaseen E.M., Dobel A.A., Albashri J.S., Alalmaei O.M. (2024). Interventions for great saphenous vein insufficiency: a systematic review and network meta-analysis. Vascular.

